# Life-threatening hemorrhagic shock due to intercostal artery perforation from a protruding staple leg after pulmonary wedge resection with a reinforced stapler: a case report

**DOI:** 10.1186/s44215-026-00256-3

**Published:** 2026-06-08

**Authors:** Shin-nosuke Watanabe, Daisuke Kimura, Takahiro Sasaki, Shuta Kimura, Chisaki Ichinohe, Tsubasa Kato, Shiori Ono, Hirose Imai, Masahito Minakawa

**Affiliations:** Department of Thoracic and Cardiovascular Surgery, Graduate School of Medicine, Hirosaki University, Hirosaki, Aomori, 5 Zaifu-cho, 036-8562 Japan

**Keywords:** Pulmonary wedge resection, Staple line reinforcement, Intercostal artery injury, Stapler-related complication, Hemorrhagic shock

## Abstract

**Background:**

Pulmonary wedge resection is a lung-sparing procedure, with mechanical staplers enhancing surgical safety. Staple line reinforcement reduces air leakage; however, its effect on preventing mechanical vascular injury remains unclear. Staple-related vascular injuries rarely cause life-threatening complications. Here, we report a rare case of hemorrhagic shock caused by intercostal artery perforation from a protruding staple leg following thoracoscopic wedge resection with a reinforced stapler, which was likely triggered by lung collapse and re-expansion.

**Case presentation:**

A 76-year-old man underwent thoracoscopic wedge resection of right segment 6 for suspected metachronous lung adenocarcinoma using a reinforced stapler with a gold cartridge. The surgery was uneventful. On postoperative day (POD) 1, accidental chest drain removal caused lung collapse. After drain reinsertion and lung re-expansion on POD 2, the patient developed hemorrhagic shock on POD 3. Emergency thoracotomy revealed massive intrathoracic hemorrhage from the eighth intercostal artery due to a staple leg protruding through the reinforcement and perforating the artery during lung inflation. Hemostasis was achieved using electrocautery and artery ligation, followed by removal of the protruding staple and additional staple line reinforcement. The postoperative course was uneventful, and the patient was discharged on POD 10.

**Conclusions:**

Mechanical vascular injury due to staple protrusion can occur despite reinforced staplers. Lung collapse and re-expansion may alter the spatial relationship between the staple line and adjacent chest wall vessels, thereby increasing arterial injury risk. Careful staple line inspection, appropriate cartridge selection based on tissue thickness, and chest drain management are essential for preventing this rare complication.

## Background

Pulmonary wedge resection is an essential lung-sparing procedure widely used for diagnosis and treatment. Advances in thoracoscopic techniques and stapling devices have improved safety and feasibility [1, 2]. Although mechanical staplers are reliable, rare staple line–related vascular injuries can cause serious and potentially life-threatening complications. However, the mechanisms and predisposing factors that underlie these injuries remain unclear. Here, we report a case of hemorrhagic shock caused by intercostal artery perforation from a protruding staple leg following pulmonary wedge resection using a reinforced stapler, precipitated by postoperative lung collapse and re-expansion. This case highlights the potential risk of staple-related mechanical vascular injury, despite reinforcement, and provides insights into potential triggering mechanisms.

## Case presentation

**Fig. 1:**
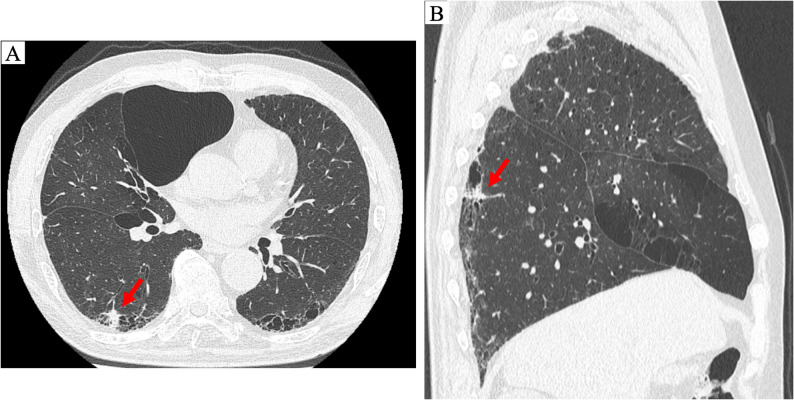
Preoperative computed tomography images. **A** Axial computed tomography (CT) image shows a 14 × 10 mm solid subpleural nodule in segment 6 of the right lower lobe (red arrow). **B** Sagittal CT image demonstrates the same lesion located adjacent to the pleural surface (red arrow)

A 76-year-old man was diagnosed with right upper lobe lung adenocarcinoma approximately five years and six months previously (cT4N3M1c, Stage IVB, TNM eighth edition, with pleural dissemination, bilateral adrenal metastases, intra-abdominal lymph node metastases, and splenic metastasis). Companion diagnostic testing revealed a *KRAS* G12C mutation and a PD-L1 tumor proportion score of 100%. The patient initially received three cycles of combination therapy (cisplatin, pemetrexed, and pembrolizumab), which was discontinued because of grade 3 diarrhea according to the Common Terminology Criteria for Adverse Events. Pembrolizumab monotherapy was resumed for eight cycles, but was discontinued owing to grade 2 immune-related pneumonitis and skin rash. The patient was treated with prednisolone (initial dose: 20 mg/day), which was gradually tapered and discontinued over several months. A complete response was maintained for approximately four years without further treatment. Follow-up computed tomography (CT) performed four years after discontinuing immune checkpoint inhibitor therapy revealed a 14 × 10 mm solid subpleural nodule in segment 6 of the right lower lobe (Fig. 1A, B). Positron emission tomography-CT demonstrated increased uptake in the lesion (maximum standardized uptake value: 4.7). Oligometastasis or metachronous primary lung cancer was suspected, and the patient was referred to our department. Considering his long-term survival from stage IVB disease and his history of immune-related pneumonitis, a minimally invasive diagnostic approach was prioritized, and thoracoscopic wedge resection was planned.

**Fig. 2:**
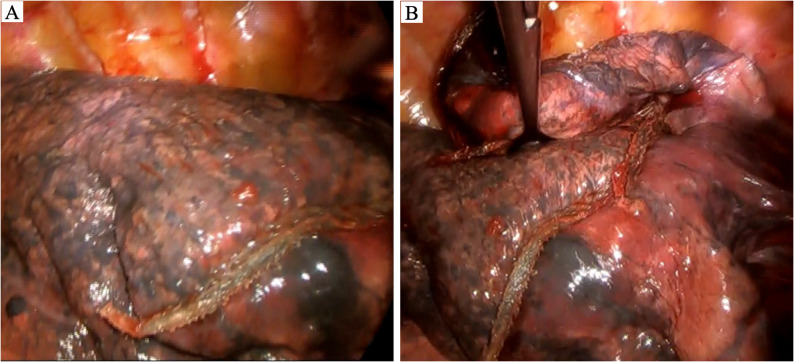
Intraoperative findings after pulmonary wedge resection using a reinforced stapler (**A**, **B**). The staple line demonstrates intact staple formation without malformation immediately after firing using the ECHELON ENDOPATH™ Staple Line Reinforcement system (60 mm, gold cartridge). Air leakage is not observed during the sealing test.

Surgery was performed under general anesthesia with single-lung ventilation in the left lateral decubitus position. A 12 mm port was placed in the seventh intercostal space along the anterior axillary line, and the S6 lesion was identified. A utility incision was made in the sixth intercostal space along the posterior axillary line. The lung parenchyma appeared thin and compliant. The cartridge was selected based on intraoperative visual and tactile assessment, taking into account the use of reinforcement material. To minimize air leakage, wedge resection was performed using the ECHELON ENDOPATH™ Staple Line Reinforcement system (60 mm, gold cartridge; Ethicon, Inc., Somerville, NJ, USA). Staple formation was satisfactory, and careful inspection of the staple line immediately after parenchymal division and during the sealing test revealed no air leakage, protruding staples, or other abnormalities (Fig. 2A, B). A chest drain was placed. After resuming bilateral lung ventilation, the staple line was re-evaluated, and no abnormalities were identified. The surgery was completed. Histopathological examination confirmed a metachronous primary lung adenocarcinoma, distinct from the previous tumor. No interstitial lung disease was observed.

On postoperative day (POD) 1, the patient became agitated and accidentally removed the chest drain. The insertion site was immediately closed, and chest radiography revealed mild subcutaneous emphysema without lung collapse. On POD 2, follow-up chest radiography demonstrated progression of subcutaneous emphysema, suggesting persistent air leakage, and a chest drain was reinserted, resulting in successful lung re-expansion. On POD 3, the patient suddenly lost consciousness and developed shock (heart rate: 110 beats/min, systolic blood pressure: 80 mmHg). Rapid blood drainage (500 mL) was observed through the chest tube. Despite a hemoglobin level of 10.5 g/dL, acute hemorrhagic shock was suspected based on hemodynamic instability, as hemoglobin levels may not immediately decrease in the early phase of acute blood loss, prompting emergency surgery. During anesthesia induction, the patient developed pulseless electrical activity due to hypovolemic shock. Cardiopulmonary resuscitation, including adrenaline administration and rapid blood transfusion, was initiated. Return of spontaneous circulation was achieved after 4 min of resuscitation, and an emergency thoracotomy was performed.

**Fig. 3:**
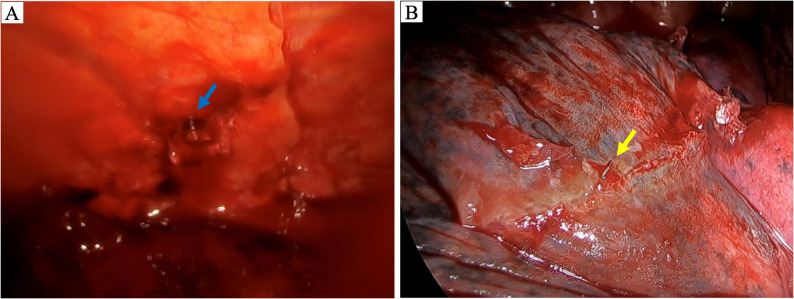
Intraoperative findings at emergency thoracotomy. **A** Active pulsatile bleeding from the eighth intercostal artery after evacuation of the intrathoracic hematoma (blue arrow). **B** A single staple leg protrudes from the staple line (yellow arrow). Lung inflation demonstrates that the protruding staple is in direct contact with the bleeding site on the eighth intercostal artery

Large hematomas were predominantly observed in the posterior basal region. After evacuation, active pulsatile bleeding from the eighth intercostal artery was observed (Fig. 3A). Hemostasis was temporarily achieved using electrocautery. Further examination revealed no pleural adhesions. A staple leg protruded from the staple line, and lung inflation demonstrated that the protruding staple was in direct contact with and capable of penetrating the bleeding site on the eighth intercostal artery (Fig. 3B). The arterial walls showed no inflammation, fragility, or macroscopic abnormalities. The protruding staple was removed, and the visceral pleural defect was repaired by suturing. Although no air leakage was detected during the sealing test, the staple line was reinforced with a polyglycolic acid sheet (Neoveil; Gunze, Kyoto, Japan) and fibrin glue. The eighth intercostal artery was ligated proximally and distally to ensure complete hemostasis. Total intraoperative blood loss, including the evacuated hematoma, was 940 mL. The postoperative course was uneventful, and the patient was discharged on POD 10.

## Discussion

Pulmonary wedge resection is a minimally invasive lung-sparing procedure, and technological advances have significantly improved its safety [1, 2]. Postoperative hemorrhage remains a serious complication and may be life-threatening [3]. Although the most common sources of bleeding include the lung parenchyma and pulmonary or bronchial vessels, injuries to the intercostal arteries have been reported [3, 4]. Direct mechanical injury to the intercostal artery caused by staple protrusion is extremely rare. Previous reports have described severe complications, such as intercostal artery injury, delayed hemothorax, and aortic injury caused by protruding staples [4–8]. These findings underscore that the mechanical interaction between staple tips and adjacent structures represents an important but underrecognized risk. Previous reports suggest that vascular injury caused by protruding staples is mainly due to continuous mechanical irritation or friction, often presenting as delayed hemothorax several days after surgery.

In the present case, a staple leg penetrated the reinforcement and perforated the eighth intercostal artery, causing hemorrhagic shock on POD 3. Although similar cases have been reported [8], previous reports have described delayed hemothorax and vascular injury caused by protruding staples; however, most cases did not involve the use of reinforced staplers. To our knowledge, this is one of the few reports demonstrating intercostal artery injury caused by a staple penetrating reinforcement material, highlighting that reinforcement does not completely preclude mechanical complications. Although reinforced staplers are generally considered safe, various complications associated with their use have been described [9]. Our case underscores that even with reinforcement, the risk of mechanical vascular injury remains a critical consideration.

Reoperation revealed no pleural adhesions or vascular wall abnormalities, suggesting direct mechanical trauma from the protruding staple. A key contributing factor was the sudden change in lung position caused by postoperative lung collapse following accidental chest tube removal, followed by rapid re-expansion. This abrupt positional shift may have brought the protruding staple into direct contact with the intercostal arteries. Because of its greater mobility, the lower lobe may be susceptible to such mechanical interactions. In compliant lung parenchyma, the staple line may assume an angular or tent-like configuration, increasing the possibility of a localized staple protrusion.

Cartridge selection may have contributed to the protrusion. Here, a gold cartridge was selected based on visual and tactile assessment of the thin parenchyma. However, the addition of reinforcement material can increase the effective tissue thickness, potentially leading to incomplete staple formation and insufficient leg bending. Although the staples may appear adequate intraoperatively, these subtle defects can manifest postoperatively as protrusions. Furthermore, respiratory motion and lung re-expansion can alter the staple line configuration or cause delayed deformation, increasing the risk of contact with adjacent structures. Notably, this complication occurred despite the use of a reinforced stapler, suggesting that reinforcement does not fully prevent such delayed mechanical changes. Therefore, considering the combined thickness of the lung tissue and reinforcement in selecting the cartridge height may help reduce this risk. Conversely, excessively high cartridges may increase the risk of air leakage, emphasizing the importance of careful intraoperative assessment and appropriate cartridge selection. These considerations are consistent with previous reports on stapler–tissue interactions, which emphasize that appropriate staple formation depends on the interplay between tissue thickness, compression, and staple height, rather than on visual thickness alone. In clinical practice, however, cartridge selection is largely based on subjective visual and tactile assessment, which may not accurately reflect the actual compressed tissue thickness at the time of stapling, particularly when additional materials such as reinforcement are used. This discrepancy may result in unrecognized staple malformation despite an apparently satisfactory intraoperative appearance [10].

Based on these observations, careful intraoperative inspection of the entire staple line is essential, even when using reinforced staplers. Particular attention should be paid to staple lines located near the chest wall or in highly mobile lung regions such as the lower lobe, where trimming of suspicious protrusions or additional coverage should be considered as preventive measures. Furthermore, as postoperative lung collapse and re-expansion can increase the risk of contact between the staples and the chest wall, vigilant postoperative management including cautious chest drain care is important to avoid abrupt lung volume changes.

## Conclusions

Life-threatening intercostal artery injury can occur because of a protruding staple leg, despite the use of reinforced staplers. Reinforcement materials do not completely eliminate the risk of mechanical vascular injury. Postoperative lung collapse and subsequent re-expansion may alter the spatial relationship between the lung and the chest wall, facilitating contact between the staple line and adjacent vessels. Careful intraoperative inspection of the staple line and appropriate cartridge selection, considering the combined thickness of the lung tissue and reinforcement material, are essential. Furthermore, vigilant postoperative chest drain management is vital to avoid sudden positional changes that may trigger rare but serious complications.

## Data Availability

Data sharing is not applicable to this article, as no datasets were generated or analyzed in the current study.

## References

[1] Ginsberg RJ, Rubinstein LV. Randomized trial of lobectomy versus limited resection for T1 N0 non-small cell lung cancer. Lung Cancer Study Group. Ann Thorac Surg. 1995;60(3):615–22. 10.1016/0003-4975(95)00537-u. discussion 622-3. 7677489 10.1016/0003-4975(95)00537-u

[2] McKenna RJ Jr, Houck W, Fuller CB. Video-assisted thoracic surgery lobectomy: experience with 1,100 cases. Ann Thorac Surg. 2006;81(2):421–5. 10.1016/j.athoracsur.2005.07.078. discussion 425-6. 16427825 10.1016/j.athoracsur.2005.07.078

[3] Yano M, Numanami H, Akiyama T, Taguchi R, Furuta C, Haniuda M. Reoperation for postoperative bleeding following pulmonary resection: a report of a single-center experience. Gen Thorac Cardiovasc Surg. 2019;67(7):608–14. 10.1007/s11748-018-01060-9. 30656590 10.1007/s11748-018-01060-9

[4] Motoyama H, Yamashina A, Chihara K. Sudden hemothorax following lobectomy caused by staple. Interact Cardiovasc Thorac Surg. 2009;9(1):117–8. 10.1510/icvts.2009.203786. 19380338 10.1510/icvts.2009.203786

[5] Yamaji M, Yano M, Okamoto S, Fukui T. Postoperative aortic injury caused by a staple line formed during wedge resection of the lung. Interact Cardiovasc Thorac Surg. 2022;35(6):ivac275. 10.1093/icvts/ivac275. 36420988 10.1093/icvts/ivac275 PMC9987216

[6] Negishi H, Tsubochi H, Maki M, Endo S. Incidental haemothorax after sublobar resection: did staple line scratch chest wall? J Surg Case Rep. 2019;2019(12):rjz276. 10.1093/jscr/rjz276. 31850143 10.1093/jscr/rjz276 PMC6908458

[7] Yamashita T, Asai K. Delayed severe hemothorax caused by a staple line of a bullectomy performed 11 years earlier. Surg Case Rep. 2023;9(1):194. 10.1186/s40792-023-01775-9. 37932485 10.1186/s40792-023-01775-9 PMC10627997

[8] Fang J, Liu Q, Wu C, Li J. Massive chest wall bleeding 3 days after lung wedge resection caused by a protruding staple. J Surg Case Rep. 2024;2024(10):rjae620. 10.1093/jscr/rjae620. 39403671 10.1093/jscr/rjae620 PMC11473127

[9] Suyama Y, Nakada T, Watanabe Y, Nakashima M, Otani A, Tsukamoto Y, Shibazaki T, Kinoshita T, Ohtsuka T. A Case of Postoperative Pneumothorax due to a Dislodged Reinforced Staple Fragment After Thoracoscopic Segmentectomy: Recommendations for the Safe Use of Reinforced Staplers. Asian J Endosc Surg. 2025 Jan-Dec;18(1):e70173. 10.1111/ases.70173. 10.1111/ases.70173 41178365

[10] Chekan E, Whelan RL. Surgical stapling device-tissue interactions: what surgeons need to know to improve patient outcomes. Med Devices (Auckl). 2014;7:305–18. 10.2147/MDER.S67338. 25246812 10.2147/MDER.S67338 PMC4168870

